# The development of inpatient cost and nursing service weights in a tertiary hospital in Malaysia

**DOI:** 10.1186/s12913-020-05776-4

**Published:** 2020-10-14

**Authors:** Nor Haty Hassan, Syed Mohamed Aljunid, Amrizal Muhammad Nur

**Affiliations:** 1 International Centre for Casemix and Clinical Coding, Faculty of Medicine, National University of Malaysia, UKM Medical Centre, Bandar Tun Razak, Cheras, 56000 Kuala Lumpur, Malaysia; 2 United Nations University – International Institute for Global Health, UKM Medical Centre, Bandar Tun Razak, Cheras, 56000 Kuala Lumpur, Malaysia; 3 Department of Nursing, Faculty of Medicine, Jalan Yaacob Latif, Bandar Tun Razak Cheras, 56000 Kuala Lumpur, Malaysia; 4 Department of Health Policy and Management, Faculty of Public Health, Kuwait University, P.O Box 24923, 13110 Safat, Kuwait

**Keywords:** Nursing costs, Nursing hours, Nursing service intensity, Service weights, Inpatient care

## Abstract

**Background:**

The current healthcare sector consists of diverse services to accommodate the high demands and expectations of the users. Nursing plays a major role in catering to these demands and expectations, but nursing costs and service weights are underestimated. Therefore, this study aimed to estimate the nursing costs and service weights as well as identify the factors that influence these costs.

**Methods:**

A retrospective cross-sectional descriptive study was conducted at Universiti Kebangsaan Malaysia Medical Centre (UKMMC) using 85,042 hospital discharges from 2009 to 2012. A casemix costing method using the step-down approach was used to derive the nursing costs and service weights. The cost analysis was performed using the hospital data obtained from five departments of the UKMMC: Finance, Human Resource, Nursing Management, Maintenance and Medical Information. The costing data were trimmed using a low trim point and high trim point (L3H3) method.

**Results:**

The highest nursing cost and service weights for medical cases were from F-4-13-II (bipolar disorders including mania - moderate, RM6,129; 4.9871). The highest nursing cost and service weights for surgical cases were from G-1-11-III (ventricular shunt - major, RM9,694; 7.8880). In obstetrics and gynaecology (O&G), the highest nursing cost and service weights were from O-6-10-III (caesarean section - major, RM2,515; 2.0467). Finally, the highest nursing cost and service weights for paediatric were from P-8-08-II (neonate birthweight > 2499 g with respiratory distress syndrome congenital pneumonia - moderate, RM1,300; 1.0582). Multiple linear regression analysis showed that nursing hours were significantly related to the following factors: length of stay (β = 7.6, *p* < 0.05), adult (β = − 6.0, *p* < 0.05), severity level I (β = − 3.2, *p* < 0.05), severity level III (β = 7.3, *p* < 0.05), male gender (β = − 4.2, *p* < 0.05), and the elderly (β = − 0.5, *p* < 0.05).

**Conclusions:**

The results showed that nursing cost and service weights were higher in surgical cases compared to other disciplines such as medical, O&G and paediatric. This is possible as there are significant differences in the nursing activities and work processes between wards and specialities.

## Background

Nursing services, the largest component of inpatient care, is a continuous activity from patients’ admission until their discharge from the hospital [1]. It is a 24-h service with a heavier workload during the day compared to the night. Nurses spend almost 60% of their time with patients in critical wards and 54% in the medical and surgical wards [2]. Furthermore, the contribution of nurses is about 30% of the overall hospital costs and 44% of nursing care costs for inpatients [3]. Most of the public tertiary hospitals in Malaysia are implementing a traditional method of hospital payment, which is based on a ‘room and board’ rate. This is presuming that the amount of nursing care is equally distributed among all patients, making the cost of nursing care an unseen variable [4]. According to Thompson, the ‘room and board’ charges are the cost of routine nursing care combined with hospital hotel costs consisting of dietary, housekeeping and linen [4]. Laport et al., define the ‘room and board’ charges as an average nursing cost per day whereby the total nursing costs are divided by the total number of inpatient days [5].

According to the billing system of Universiti Kebangsaan Malaysia Medical Centre (UKMMC) in 2011, the room charge for an adult was RM40 and charge of treatment was RM50. In addition, the room charge for paediatric is RM15 and charge for treatment was RM25. Additionally, there is a charge if any extra investigation or treatment was rendered to the patient. These data on hospital charges in UKMMC showcased that the charges are only differentiated according to adult or paediatrics categories and they increase with the length of hospital stay. These types of charges neglect the complexity of the illness which is the reason for the increase in the cost as there was a need for more nursing care. Therefore, the traditional method of hospital payment could result in either under- or over-charging of nursing services, which could contribute to the improper hospital reimbursement.

A study by Welton and Fischer compared the hospital charges, based on room and board rate and nursing service intensity [6]. The study found that charges which were based on nursing service intensity were more accurate and reduced the risk of underestimating the nursing cost [6]. Another factor, which influences the hospital cost and charges, is the employment of registered nurses with a bachelor’s degree. Currently, the minimum requirement for a nurse in Malaysia is a Diploma. However, after the recent phenomena in Malaysian healthcare, the Ministry of Health Malaysia stated that future nurses must hold a minimum of bachelor’s degree in nursing. This may raise the nursing costs, as payment for registered nurses’ salaries with a bachelor’s degree will require a higher portion of the hospital budget. This was proven by Welton and Fischer who discovered the differences in cost of using services of a mixed category of nurses [7]. The contribution of registered nurses on salary and hours of care can be as high as 25.5% of hospital expenditures annually [8]. This is in line with the findings of a study by Mark et al., which stated that costs were increased when many registered nurses were hired in the hospital [9]. Nonetheless, although the salary of registered nurses was high and found to contribute to the high cost of treatment, knowledgeable and highly skilful nurses are important for a positive patient outcome [10].

Taking into consideration the major contribution of nursing services for inpatient care, in addition to the increase of hospital costs and limited healthcare resources, nursing researchers began to study the relationship between nursing services and hospital costs [5, 7]. Many countries worldwide have progressively conducted nursing cost calculations with variations in study samples and output [11].

The nursing services cost can have multiple meanings, as it will be based on methodology and results [12]. The nursing cost could be measured in several ways such as cost per patient, cost per day, patient care per hour, acuity-adjusted per patient day, average nursing wage, nursing hours or per visit as well as by using diagnosis-related group (DRG) [12–14]. DRG is a patient care classification system that was introduced in 1983 for the inpatient prospective payment system, an addition to the Medicare system in the United States [15]. It is a health management tool that categorises patients according to their similarities in clinical information and the healthcare resources used [15–18].

Service weights are related to DRG and describe the costs according to it for a particular service [19]. It is defined as the mean cost of specific services for any patient type, relative to the mean cost of all patient types [17]. Service weights are used to measure common resources consumed by patients in each DRG [5]. For example, DRG with a service weight of 4.0 is a service that consumed four times more resources than DRG with a service weight of 1.0 [20]. It is useful to obtain information on the budget spent on patients in each DRG [19].

Other similar terms with service weights are service intensity weights, service cost weights and relative weights [20–22]. Nursing intensity weights were created to adjust the relative weights of each DRG for hospital reimbursement and adjustment of payment system to reflect nursing resources [20, 23]. Relative weights have been used to construct payment based on DRG, using the formula of comparing the mean cost of a DRG with the mean cost for all DRGs [1]. The definition of a cost weight is the average cost of a DRG level compared to the average cost across all DRGs [5]. Specific components of cost weights are known as service weights or ratios, and are used for nursing, laboratory and radiology costs [24].

Knauf et al. found that cardiology cases have an average nursing intensity weight of 3.11 and orthopaedic cases have an average nursing intensity weight of 2.26 [1]. Apart from merely reporting the crude cost weights, it is also crucial to determine if there were ‘overestimation’ or ‘underestimation’ of the cost weights. Overestimation occurs when the estimated cost weight is higher than the actual cost weight. In contrast, underestimation is when the estimated cost weight is lower than the actual cost weight [24].

There were limited studies in Malaysia, which determined the cost weights for treatment and services. However, a few studies have been carried out in UKMMC to determine the cost weights across other disciplines. For instance, the output of a study on cost weights for treatment of cardiology showed that the range of cost weights was between 0.4710 and 0.7961, while the cost weights for orthopaedic cases were between 0.7438 and 1.8182 [25]. A study done by Ali Jadoo et al. to determine the service weights for pharmacy showed that the range of pharmacy service weights was between 3.78 and 11.80 [26]. In a recent study by Roszita on the laboratory and radiology service weights, the laboratory service weights was between 1.6896 and 5.9609, while radiology service weights was between 1.6336 and 2.8461 [27].

As there is a lack in the study on the estimation of nursing costs and service weights in Malaysia, this study aimed to estimate the nursing resources used by inpatients in UKMMC and further review the factors influencing nursing cost in UKMMC. In this study, the calculation of nursing cost was linked with the Malaysia Diagnosis Related Group (MY-DRG®) casemix system.

## Methods

### Study population and designs

A cross-sectional study was conducted to determine the inpatient nursing costs and service weights in UKMMC as well as factors associated with nursing costs from November 2012 to August 2014. UKMMC is a public teaching hospital with 1000 beds owned by the Ministry of Education [28]. It is one of the premier teaching hospitals in Malaysia providing secondary and tertiary levels of inpatient and outpatient services. The hospital also serves as a referral hospital to secondary level hospitals in various parts of Malaysia. In 2002, UKMMC became the first medical centre in Malaysia to introduce the casemix system for managing their patients. To date, in UKMMC, casemix system is being used for research purposes and not as the provider payment mechanism. The main purpose of its development was to enhance the quality and efficiency of hospital services. UKMMC uses MY-DRG® grouper, which is the customised version of the United Nations University-Case Based Group (UNU-CBG) casemix system [29]. MY-DRG® is a patient classification system, which belongs to the casemix system and is being used in Malaysia. It was developed by a team of researchers at the United Nations University, International Institute for Global Health and the International Casemix Centre and Clinical Coding. The MY-DRG® grouper software is used to categorise the cases into various mutually exclusive casemix groups using the minimum data set of each patient. MY-DRG® consists of 1220 groups, which can be extended according to the needs of the country. Each group was organised into five alphanumeric codes (one letter and four numbers).

The first digit refers to casemix main groups (CMGs). CMGs are the first level of classification labelled with the alphabet (A to Z). Generally, it is equivalent to chapters in ICD-10 and corresponds to body systems and payment packages. Within MY-DRG**®**, it comprises 31 CMGs which include 22 CMGs which refer to acute care CMGs, two ambulatory CMGs, two sub-acute and chronic CMGs, four special CMGs and one error CMGs. Currently, UKMMC has implemented 24 CMGs including one error. CMG gives wider options to use diagnosis, procedures, drugs, investigations and the prosthesis in the classification [30, 31]. In the MY-DRG® casemix system, case-type is defined based on four major disciplines in the hospital. The case-type is represented in the second digit of the MY-DRG® code. Case-type 1 is surgical inpatient cases, case-type 4 is medical inpatient cases, case-type 6 is O&G inpatients cases and case-type 8 is paediatric inpatient cases. The surgical case-type, case-type 1, covers all cases that underwent surgical procedures while cases that did not have any surgical procedures are grouped into case-type 4 and referred to as medical cases. The four case-types covered all the disciplines in the hospitals including general surgery, orthopedic, psychiatric and ophthalmology.

In MY-DRG® casemix system, cases are grouped into one of the three severity levels based on the co-morbidity and complications. Severity level I is a case without co-morbidity and complication. Severity level II is a case with mild co-morbidity and complication and severity level III is a case with severe co-morbidity and complication. Hence, severity level I cases are less severe compared to cases in severity level II and III whereas Severity level II cases are less severe compared to level III.

As the main objective of this study is to determine the nursing cost and service weights for UKMMC, the data collection of this study involved data retrieved from only UKMMC. This is a crucial study as till date other ancillary services in UKMMC such as pharmacy, laboratory and radiology have already determined their service costs and service weights.

The universal sampling method was used in this study. All hospital discharges in casemix system database from 2009 to 2012 and groupable when using MY-DRG® grouper were included as study samples. The total study samples comprised of 90,581 discharge cases and were grouped into 708 MY-DRG® grouping software.

### Data collection tool

A UKMMC costing template three main components were used:

- Costing template included the number of nurses, patient days and discharges, annual staff salary, operating costs and the total number of equipment purchased in the last 5 years (the useful life of the equipment was assumed to be 5 years).
- Definitions (description of each terminology in UKMMC) such as cost centres in UKMMC.
- Basic data consisting of data on hospital performance, structure, and staffing.

Five departments of UKMMC were involved in data collection, namely: Finance, Nursing Management, Human Resources, Health Information, and Maintenance department. Hospital information data for 2011 were compiled to complete the costing template.

The costing template was distributed to all the departments. They were required to complete the hospital information according to their respective departments.

- Finance Department: total expenditure on purchased items in the last 5 years, operating cost and annual staff salary;
- Nursing Management Department: number of nurses;
- Human Resources department: number of staff;
- Health Information Department: number of inpatient days, number of discharges, number of inpatients and the average length of stay (ALOS);
- Maintenance Department: measurement of hospital floor area.

This information was essential to assist in the step-down costing and calculation of nursing costs and service weights.

### Analysis

#### Data analyses

Data were collected and transferred into a database using Statistical Package for the Social Sciences (SPSS) version 22 for costing and statistical analyses. The descriptive analysis was used for cost analyses. Furthermore, multivariate analysis using multiple linear regressions was carried out to determine factors that might influence the nursing costs. All analyses were done using IBM SPSS version 22 and a *p*-value of < 0.05 was considered as statistically significant.

#### Costing analyses

The step-down costing method was used for this study. For step-down costing, seven steps were applied to calculate the components of overhead, intermediate and final cost centres of medical, surgery, O&G and paediatric disciplines for the study. The seven steps adopted from Shepard et al. were as follows: identifying total expenditure of the hospital, classifying hospital departments by cost centres, selecting statistics to use as allocation bases, identifying total cost by individual cost centres, allocating the costs of overhead cost centres to intermediate cost centres, allocating the costs of intermediate cost centres to final cost centres, and after estimated costs have been allocated using step-down costing, relating cost to casemix group, DRG [32].

Nursing hours were used as a basic component in the calculation of nursing costs. There were eight steps to follow to develop the nursing cost:

- Step 1: Calculation of nursing hours per year per nurse. In the first step, the calculation of nursing hours was based on allocated hours according to the work shifts of the nurses.
- Step 2: Calculation of nursing hours per patient per day of the hospital stay of each cost centre or discipline is the nursing hours per year for each cost centre or discipline divided by inpatient days. The pertinent information required in this step is the number of nurses, the number of inpatient days for each cost centre or discipline and ALOS for each inpatient in the cost centre or discipline.
- Step 3: Data trimming: The purpose of data trimming is to handle the outliers before proceeding to the next step of the calculation. The term ‘inlier’ was used for cases that are treated within the standard length of stay (LOS). The definition of standard LOS is a low trim point and a high trim point, between which the average treatment cases are expected to be located [23]. A low trim point for MY-DRG® is one third multiplied with an average length of stay for MY-DRG®. A high trim point for MY-DRG® is three multiplied with the ALOS for MY-DRG®. This formula indicated that those cases with less than one-third of the ALOS are low outliers and for cases with LOS, more than three times ALOS are defined as high outliers [33]. It is to be noted that, trimming is only done in cases for MY-DRG® when (n) are more than 30 and for the group (n) less than 30, the extreme length of stay will be considered as outliers and should be eliminated [11]. As a result there were 85,042 cases with 704 MY-DRG® included in the costing analysis.
- Step 4: Calculation of nursing hours for each MY-DRG®: The fourth step was the calculation of nursing hours for each MY-DRG® is the nursing hours per patient per day of stay for each cost centre or discipline multiplied by LOS for each case in MY-DRG®.
- Step 5: Calculation of mean nursing hours for each MY-DRG®: To calculate the mean nursing hours for each MY-DRG®, the total number of nursing hours per MY-DRG® is divided by the total number of frequency per MY-DRG®.
- Step 6: Calculation of nursing service weights per MY-DRG® is the mean nursing hours per MY-DRG® divided by total mean nursing hours for all MY-DRGs®.
- Step 7: Calculation of hospital nursing based rate (HNBR): HNBR was essential to determine first because the results would be required to calculate the nursing costs. The formula used is total nursing expenditure for inpatients divided by the total of discharges.

Nursing expenditure for inpatients is the total nursing expenditure which means the Department of Nursing Management expenditure inclusive of emolument (total of nurses’ salaries for inpatients) + 70% of the total cost of operational + 70% total cost of equipment (total cost of equipment/4.329 – is a discount rate). In this study, the rationale of 70% was chosen because it was assumed that 70% of the expenditures were involved for the inpatients and 30% for outpatients.

- Step 8: Calculation of nursing cost per MY-DRG®: To calculate the nursing cost per MY-DRG®, the nursing service weights per MY-DRG® were multiplied by HNBR

## Results

The trimming process using L3H3 method was used to include and exclude the data for analyses. The final total of discharged inpatients of this study comprised 85,042 included cases and 5539 were excluded cases in the costing analyses.

### Highest nursing cost according to discipline

Based on MY-DRG® casemix system, discharged inpatient cases were categorised into three levels of severity. Severity I refers to patients who do not have complications and co-morbidities. Patients in severity level II have mild complications and co-morbidities and those in severity level III have severe complications and co-morbidities.

The output from the costing analysis shows that the highest nursing cost and service weight was recorded among the surgical discipline cases wherein the highest nursing cost amounted to RM9,694 and service weight was 7.8880. This highest nursing cost and service weight recorded under MY-DRG® was G-1-11-III (ventricular shunt – major). The highest nursing cost and service weight recorded under surgical discipline was almost nine times higher than the highest nursing cost and service weight recorded under paediatrics’ cases. The costing analysis revealed that the highest nursing cost across paediatric discipline was only RM1,300 with a service weight of 1.0582. This cost recorded using MY-DRG® was P-8-08-II (neonate birthweight > 2499 g with respiratory distress syndrome and congenital pneumonia – moderate).

This following sub-topic presents the top five highest nursing costs and service weights according to the four disciplines under MY-DRG® namely, medical, surgical, O&G and paediatric.

#### Medical

The top five highest nursing costs and service weights in the medical discipline are shown in Table 1. As seen in the table, four of the cases were from CMG ‘F’ (mental health and behavioural group). The MY-DRG® with the highest nursing cost and service weight was F-4-13-II (bipolar disorders including mania - moderate) with the nursing cost of RM6,129 and service weight (NSW) of 4.9871.

**Table 1:** Top five highest costs and nursing service weights for medical discipline

MY-DRG®	MY-DRG® description	Average length of stay	Mean nursing hours	Nursing cost (RM)	Nursing service weights
F-4-13-II	Bipolar disorders including mania - moderate	23.09	205.47	6129	4.9871
F-4-10-I	Schizophrenia - mild	20.81	176.08	5252	4.2737
F-4-10-II	Schizophrenia - moderate	20.71	165.92	4949	4.0272
F-4-13-I	Bipolar disorders including mania - mild	18.56	158.82	4737	3.8549
Z-4-12-III	Other factors influencing health status - severe	26.15	154.11	4, 597	3.7405

#### Surgical

The top five highest nursing costs and service weights in the surgical discipline consisted of different types of cases, all of which were from severity level III. It is seen from Table 2 that the MY-DRG® with the highest nursing cost and service weight was G-1-11-III (ventricular shunt - major) with the nursing cost of RM9,694 and a service weight of 7.8880.

**Table 2:** Top five highest costs and nursing service weights for surgical discipline

MY-DRG®	MY-DRG® description	Average length of stay	Mean nursing hours	Nursing cost (RM)	Nursing service weights
G-1-11-III	Ventricular shunt - major	37.09	324.99	9694	7.8880
D-1-10-III	Bone marrow & stem cell transplant - major	35.20	313.93	9364	7.6196
L-1-30-III	Skin graft excluding burns - major	33.16	293.75	8762	7.1299
M-1-03-III	Spinal fusion procedures - major	30.64	272.22	8120	6.6074
U-1-20-III	Other ear nose mouth & throat operations - major	30.23	267.35	7974	6.4890

#### Obstetrics & Gynaecology (O&G)

The top five highest nursing costs and service weights for O&G are as listed in Table 3. As seen in the table, MY-DRG® with the highest nursing cost and service weight was O-6-10-III (caesarean section - major) and with the nursing cost of RM2,515 and service weight of 2.0467.

**Table 3:** Top five highest costs and nursing service weights for obstetrics & gynaecology discipline

MY-DRG®	MY-DRG® description	Average length of stay	Mean nursing hours	Nursing cost (RM)	Nursing service weights
O-6-10-III	Caesarean section - major	6.33	84.32	2515	2.0467
O-6-10-II	Caesarean section - moderate	5.01	67.69	2019	1.6430
O-6-11-I	Vaginal delivery with fallopian destruction &/or dilation & curettage - minor	4.27	58.39	1741	1.4172
O-6-10-I	Caesarean section - minor	4.36	58.30	1738	1.4149
O-6-11-II	Vaginal delivery with fallopian destruction &/or dilation & curettage - moderate	4.16	56.20	1676	1.3640

#### Paediatric

The top five highest nursing costs and service weights by paediatrics are shown in Table 4. The nursing cost and service weight was the highest among cases that were grouped under MY-DRG® P-8-08-II (neonate birthweight> 2499 g with respiratory distress syndrome and congenital pneumonia - moderate) with the nursing cost of RM1,300 and service weight of 1.0582.

**Table 4:** Top five highest costs and nursing service weights for paediatrics discipline

MY-DRG®	MY-DRG® description	Average length of stay	Mean nursing hours	Nursing cost (RM)	Nursing service weights
P-8-08-II	Neonate birthweight > 2499 g with respiratory distress syndrome & congenital pneumonia - moderate	3.67	43.60	1300	1.0582
P-8-15-I	Neonate birthweight > 2499 g with aspiration syndrome - mild	4.25	42.10	1255	1.0218
P-8-16-II	Neonate birthweight > 2499 g with congenital/perinatal sepsis - moderate	4.25	42.05	1254	1.0206
P-8-17-III	Neonate birthweight > 2499 g with complex operation - severe	3.96	39.97	1192	0.9702
P-8-14-I	Neonate birthweight > 2499 g with complex congenital abnormalities - mild	3.73	37.43	1116	0.9084

### Factors influencing nursing costs

The multiple linear regressions (MLR) analysis was used to determine factors influencing the nursing costs. The total number of nursing hours per patient per stay was regarded as the dependent variable of the analysis. This was because nursing hours were the basic component used to obtain the nursing costs in this study. The independent variables were age, length of stay, gender, and severity level. During the multivariate analyses, the age of the discharged inpatients was further categorised into three categories: elderly ≥60 years old, paediatrics ≤12 years old and adults (between the age of elderly and paediatrics). These categories of age were used for multiple linear regression analysis to determine factors influencing nursing costs. The classification was made according to the admission policy of UKMMC, for example, for paediatric wards were for patients aged ≤12 years. MLR showed a strong relationship between the dependent variable and independent variables (0.949). Age, length of stay, severity level, and gender influenced almost all (90.1%) of the nursing hours. It was estimated that at least 10% was influenced by other factors, which were not included in this investigation. The nursing hours was significantly related to the length of stay (β = 7.6, *p* < 0.05), adults (β = − 6.0, *p* < 0.05), severity level I (β = − 3.2, *p* < 0.05), severity level III (β = 7.3, *p* < 0.05), male gender (β = − 4.2, *p* < 0.05), and elderly (β = − 0.5, *p* < 0.05). The results of multivariate analyses showed that the male group has a total of − 4.2 lower nursing hours compared to the female group. The adult group had − 6.0 lower hours compared to the paediatric group. Further, the elderly group had − 0.5 lower hours compared to the paediatric group. For the variable “severity level”, severity level III had 7.3 higher nursing hours compared to severity level two. Severity level one had − 3.2 lower hours compared to severity level II. Thus, the prediction model of this study was:

$$ \mathrm{Total}\ \mathrm{nursing}\ \mathrm{hours}\ \mathrm{per}\ \mathrm{patient}\ \mathrm{per}\ \mathrm{episode}\ \mathrm{of}\ \mathrm{stay}=0.297-\left[4.2\ \mathrm{X}\ \mathrm{male}\right]-\left[6.0\ \mathrm{X}\ \mathrm{adult}\right]-\left[0.5\ \mathrm{X}\ \mathrm{elderly}\right]+\left[7.3\ \mathrm{X}\ \mathrm{severity}\ \mathrm{level}\ \mathrm{I}\mathrm{I}\mathrm{I}\right]-\left[3.2\ \mathrm{X}\ \mathrm{severity}\ \mathrm{level}\ \mathrm{I}\right]+\left[7.6\ \mathrm{X}\ \mathrm{LOS}\right] $$

## Discussion

Nursing services can be measured in several ways. Previous studies have shown that different methods used in the calculation of nursing cost would result in different types of output. As stated by Riewpaiboon et al. and suggested by Negrini et al. the variation of results might be obtained from the different costing methods, depending on the requirement and interest of the users [34, 35]. The unstandardized method was used most probably due to the different interests and demands of the organisations and users [36, 37]. However, due to the inconsistency in the costing methodology, the different types of outputs caused difficulties in comparing the cost outcomes between healthcare settings such as hospitals [38]. Besides, the determination of service weights is also a challenging part for developing countries like Malaysia [38].

In summary, the top five highest nursing costs and service weights in the medical discipline was MY-DRG® F-4-13-II (bipolar disorders including mania - moderate) (RM6,129; 4.9871), surgical discipline was MY-DRG® G-1-11-III (ventricular shunt - major) (RM9,694; 7.8880), O&G was MY-DRG® O-6-10-III (caesarean section -major) (RM2,515; 2.0467) and paediatric was MY-DRG® P-8-08-II (neonate birthweight > 2499 g with respiratory distress syndrome and congenital pneumonia – moderate) (RM1,300; 1.0582).

This study is the first of its kind in Malaysia. Due to the insufficient nursing information on the time spent by nurses on their nursing activities, the nursing costs of this study were derived from their working hours for a year based on standard working hours of every month. From the research point of view, direct care which is more easily determined as nursing activities were documented in the nursing progress report for individual patients. In contrast, the indirect nursing care, such as telephone calls, documentation or meeting with doctors and family, is more difficult to determine, and hence, the estimated time spent on those activities were not documented [12]. Other studies used different ways for calculating the nursing costs. Upenieks et al. used the nursing time that was classified into value-added and non-value-added nursing care [2]. For developed countries such as the United States, the information on nursing activities is more advanced as they used the computerisation approach to monitor and record the nursing activities [13].

Based on the results of this study, the variability of nursing costs across DRG was feasible to be determined using mean nursing hours per MY-DRG® as the basic components of the calculation was based on nursing hours. There have been arguments against using the LOS to determine nursing costs, as the use of LOS would lead to more homogenous groups, and the significant part of the variations was not measured [11]. However, this study believed that the nursing hours per case for every MY-DRG® could be determined indirectly in the initial steps of the calculation using LOS. Finally, the variation of nursing times and nursing costs were generated for every 704 MY-DRG® comprising of 85,042 of cases. The total number of study samples was quite similar to a study done by Gong et al. describing Chinese hospital activity using Australian Casemix Diagnosis Related Group [39]. In that study, there were 84,028 records, which were grouped into 529 DRG involving three public hospitals in Chengdu within a year [39]. Study findings proved that patients with similar MY-DRG® required different amounts of time and their nursing needs also varied. Based on the nursing hours obtained per MY-DRG®, this could highlight the heterogeneity of nursing activities within DRG. The findings in this study corresponded to a study done by Pirson et al. which found that nursing activities are highly heterogeneous within DRG with coefficients of variation varying between 0.47 and 1.40 [40]. Thus, DRG was an inappropriate approach to determine the nursing intensity or variability of nursing resources consumed by each patient [41]. However, DRG is very useful to determine the variation of nursing hours across DRG [14]. The result of the study by John Thompson and Fetter on the model of counting the nursing resources within DRG found that there was high variability in nursing time from one DRG to another DRG [5]. The study findings using MY-DRG® revealed the variability of nursing costs and nursing service weights for every specific case type, namely, medical, surgical, obstetrics and gynaecology and paediatrics. Service weights reflected the resources or nursing workload used by different types of patients or illnesses. Thus service weights that are higher indicate more resources consumed [42]. By using DRG, the casemix system enables the development of nursing costs based on patient’s severity level [1].

The result of this study highlighted that the highest nursing costs and service weights for medical MY-DRG® was from F-4-13-II (bipolar disorders including mania - moderate) (RM6,129; 4.9871). Four out of five MY-DRG® were from CMG ‘F’ (mental health and behavioural groups). One of the factors influencing the nursing cost analysis was the patient’s length of stay. In this study, the longest LOS among CMGs was from CMG ‘F’ (mental health and behavioural groups), the mean length of stay was 16.50 (11.41) and maximum LOS was 63 days compared to other CMGs. The findings supported by Wilson et al. stated that hospital factors might differ from study to study, therefore, LOS is typically different in different parts of the country [42]. For example, a study done by Auffarth et al. comparing psychiatric inpatients stay in the United States and Germany hospital, found that the average inpatient stay for mental health patients were shorter in the United States compared to Germany, and this was due to the cultural differences and diversity of health system [43]. Pertaining to the illness, a study done by Addisu et al. found that patients with psychoses and bipolar disorder have longer LOS and higher cost of care compared to other cases such as depressive disorder. Furthermore, the extrapyramidal side effects due to the medication was one of the factors that contributed to the longer LOS [44]. On the other hand, for surgery cases, the highest nursing costs and service weight was from MY-DRG® G-1-11-III (ventricular shunt - major) (RM9,694; 7.8880). This result is similar to a study by Knauf et al. that showed neurosurgery cases had the highest nursing intensity weight. They also found that nursing intensity was highest at two peak times: admission and surgery [1]. Nevertheless, those results were not suitable to compare with the methodology as the classification of case types and DRG were very different from the Malaysian settings. A study by van den Oetelaar et al. in six surgical wards of academic hospital Netherland found significant differences in nursing activities between the wards. Furthermore, he stated that different specialities may have different work processes [45].

### Factors influencing nursing cost

There are limited studies available to determine factors that could potentially influence the nursing costs. The analyses done in this study were similar to the study done by O’Brien - Pallas et al. wherein nursing hours were used as the dependent variable in the regression analyses to determine factors that influence the nursing workload [46]. In this study, findings showed that the LOS, severity level, age and gender were the predictors for the nursing costs. The most useful predictor was LOS. The results showed that when LOS increased, so did the nursing hours. Nursing care needs were significantly different for each patient and the length of stay [21]. This was possible as the nursing cost increases proportionally with LOS. On the other hand, Knauf et al., showed that shortened LOS caused the greater variability of nursing care required by patients [1]. The literature reviews have highlighted that employing an experienced and educated nurse has positive patient outcomes in acute care settings and that nurses may reduce the risk of nosocomial complications among patients and reduce LOS, resulting in medical saving and better patient outcomes [10, 47]. This is inconsistent with other studies which indicated that LOS added only 5% variation [48]. Nonetheless, patient’s complexity and total LOS are the two factors that could cause the variation in nursing costs [13]. This was because more the patient days, more nursing activities will be required such as more vital signs to record, medication to administer, and daily care of personal hygiene.

In this study, the second predictor that could influence nursing costs was the severity level. Previous studies have indicated that two main factors that could cause the rise in nursing costs were: higher complexity of patient care and LOS [21]. Both factors could cause an increase in demands for nurses and their salary, decrease nurse-patient ratios and increase the variability of nursing care demands during hospital stay [5]. However, patients who are severely ill will get more attention which increases the number of nursing services. Moreover, the number of patients and patients’ severity of illness can also influence the nursing intensity or workload [14]. In a study predicting the cost for stroke patients, the factors which profoundly influenced the cost for inpatients with stroke were level of functioning after stroke, co-morbidity, complications and days of stay for non-medical reasons [49].

The third predictor was the age of inpatients. Closeness to death and the patient’s age are also factors that could cause the increase in nursing care in the acute care settings [50]. This is consistent with the findings of the present study and based on the categories of age, adults (> 12 years and < than 60 years old) and elderly (> 60 years) that will influence the nursing costs or nursing hours. However, adults and elderly showed negative value towards nursing hours compared to paediatrics.

### Limitations

The population of this study was from a single hospital, UKMMC. In Malaysia, there are only two hospitals that have implemented the casemix system for the management of patients. These hospitals are UKMMC and Universiti Sains Malaysia (USM) Hospital. However, the study did not obtain samples from USM Hospital as the main purpose of this study was to determine the nursing costs and service weights for UKMMC only. The researcher was aware that with a larger sample population, the results could be more generalised. Although the findings of this study could not be generalised for the entire country, the study output can potentially be used to benchmark other hospitals in Malaysia. However, the service weights, which refer to the ratio of nursing costs of one DRG to the overall DRG cost is an important finding that can be used to highlight the value of nursing services in hospital care.

Nursing is the major contributor in the hospital for inpatients, but the nursing resources consumed by patients were difficult to determine. Due to the insufficient of concrete data to provide the time spend by nurses to manage the individual patients under their care, this study has used the nursing hours to estimate the nursing service cost. This is in contrast to some study in developed countries where the electronic nursing database has been used to assist in estimating the nursing cost. The electronic nursing database is useful as it can include all the information related to nursing activities, increase the accuracy of the calculation of nursing times and enable to monitor the allocation of resources according to the needs of the inpatients. Further it can be customized according to the needs of the nursing services.

## Conclusion

Nursing has a major function in the management of patient care. Findings of this study indicated that nursing cost and nursing resources consumed by inpatients across different MY-DRG® can be successfully be determined using the casemix system. This study also addressed the fact that nursing cost contributes a significant portion to the hospital cost. The study showed that the higher the nursing service weights, more nursing resources and nursing workload are required to provide the inpatient care.

This study may serve as an exemplar for other extensive studies related to nursing costs. However, with the current status confronting the Malaysian economy, understandably it would not be feasible to use the nursing electronic database in the healthcare system in the near future. Therefore, as an alternative, future research is needed to refine the methods of the nursing costs and develops a better approach to economically justify the nursing resources consumed by inpatients. It is advisable that these studies be carried out in stages and by disciplines to ease in the management of the data and reduce errors. It is undeniable that UKMMC needs to develop a nursing electronic database that provides information about nursing activities for inpatients which can determine highly accurate nursing costs and service weights.

## Data Availability

The data and materials for this study are available at the International Centre for Casemix and Clinical Coding (ITCC), Faculty of Medicine, Universiti Kebangsaan Malaysia (UKM). To request these data, interested parties may seek approval by writing to the Dean of Faculty, Faculty of Medicine, Universiti Kebangsaan Malaysia, Jalan Yaacob Latif, Bandar Tun Razak, 56000 Cheras Kuala Lumpur, Malaysia.

## Abbreviations

ALOS: Average length of stay
CMG: Casemix main group
DRG: Diagnosis related group
DRGs: Diagnosis related groups
LOS: Length of stay
MLR: Multiple linear regressions
MY-DRG®: Malaysia Diagnostic Related Group
O&G: Obstetrics and gynecology
SPSS: Statistical Package for the Social Sciences
UKMMC: Universiti Kebangsaan Malaysia Medical Centre
UNU-CBG: United Nations University-Case Based Group

## References

[1] Knauf RA, Ballard K, Mossman PN, Lichtig LK (2006). Nursing cost by DRG: nursing intensity weights. Policy Polit Nurs Pract..

[2] Upenieks VV, Akhavan J, Kotlerman J, Esser J, Ngo MJ (2007). Value-added care: a new way of assessing nursing staffing ratios and workload variability. J Nurs Adm..

[3] Kane NM, Siegrist RB (2002). Understanding rising hospital inpatients: key components of cost and the impact of poor quality.

[4] Thompson JD (1984). The measurement of nursing intensity. Health Care Financing Review.

[5] Laport N, Sermeus W, Vanden Boer G, Van Herck P (2008). Adjusting for nursing care case mix in hospital reimbursement: a review of international practice. Policy Polit Nurs Pract..

[6] Welton JM, Fischer MH, DeGrace Sharon MN, Zone-Smith L (2006). Nursing intensity billing. J Nurs Adm..

[7] Rutherford MM (2008). The how, what, and why of valuation and nursing. Nurs Econ..

[8] Welton JM (2011). Hospital nursing workforce costs, wages, occupational mix, and resource utilization. J Nurs Adm..

[9] Mark BA, Lindley L, Jones CB (2009). Nurse working conditions and nursing unit costs. Policy Polit Nurs Pract..

[10] Storfjell JL, Omoike O, Ohlson S (2008). The balancing act. Patient care time versus cost. J Nurs Adm..

[11] Pirson M, Delo C, Pierdomenico LD, Laport N, Biloque V, Leclercq P (2013). Variability of nursing care by APR-DRG and by severity of illness in a sample of nine Belgian hospitals. BMC Nurs..

[12] Chiang B (2009). Estimating nursing costs - a methodological review. Int J Nurs Stud..

[13] Finkler SA, Graf CM (2001). Budgeting concepts for nurse managers.

[14] Jenkins P, Welton JM (2014). Measuring direct nursing cost per patient in the acute care setting. J Nurs Adm..

[15] Hensen P, Fürstenberg T, Luger TA, Steinhoff M, Roeder N (2005). Case mix measures and diagnosis-related groups: opportunities and threats for inpatient dermatology. J Eur Acad Dermatol Venereol..

[16] Averill RF, Muldoon JH, Vertrees JC, Goldfield NI (1998). The evolution of casemix measurement. 3M health information systems research Report.

[17] Hovenga EJS. Casemix and information systems. Health Informatics. 1996; https://www.researchgate.net/publication/265740023_Casemix_and_information_systems. Accessed 15 Apr 2017.

[18] Sanderson H, Anthony P, Mountney L (1998). Casemix for all.

[19] McKinley S (1995). Casemix update: Australian critical care costs and service weights part 1. Australian Critical Care..

[20] Welton JM, Zone-Smith L, Fischer MH (2006). Adjustment of inpatient care reimbursement for nursing intensity. Policy Polit Nurs Pract..

[21] Graf CM, Millar S, Feilteau C, Coakley PJ, Erickson JI (2003). Patients’ needs for nursing care: beyond staffing ratios. J Nurs Adm..

[22] Schreyögg J, Tiemann O, Busse R (2006). Case accounting to determine prices: how well do prices reflect costs in the German DRG-system?. Health Care Manag Sci..

[23] Keepnews DM (2006). Nursing intensity and hospital payment. Policy Polit Nurs Pract..

[24] Botz CK, Sutherland J, Lawrenson J (2006). Cost weight compression: impact of cost data precision and completeness. Health Care Financing Review/Spring..

[25] Rohaizat Y, Amrizal MN, Saperi S, Aljunid SM (2005). Cost analysis and cost weight for the treatment of orthopedic cases in a teaching hospital in Malaysia using the case-mix approach: the experience of UKM Hospital. Malaysian J Public Health..

[26] Ali Jadoo SA, Aljunid SM, Zafar A, Dexter VD (2015). Development of MY-DRG casemix pharmacy service weights in UKM medical Centre in Malaysia. DARU J Pharmaceut Sci..

[27] Roszita I (2016). Malaysia diagnosis related group (MY-DRG®): Membangunkan Pemberat Perkhidmatan Sistem Casemix untuk Ujian Makmal Diagnostik dan Prosedur Radiologi di Pusat Perubatan Universiti Kebangsaan Malaysia. Tesis Dr Falsafah.

[28] Aljunid SM, Aung YN, Ismail A, Abdul Rashid SAZ, Nur AM, Cheah J, Priya M (2019). Economic burden of hypoglycemia for type II diabetes mellitus patients in Malaysia. J PLoS One..

[29] Aljunid SM, Saperi S, Amrizal MN, Rohaizat Y (2004). HUKM Casemix system Progress report.

[30] United Nations University, International Institute for Global Health (UNU-IIGH) (2011). UNU-Grouper v1.0 (Malaysia) Software User Manual.

[31] Amrizal MN, Rohaizat Y, Zafar A, Saperi S, Aljunid SM (2005). Casemix Costing in Universiti Kebangsaan Malaysia Hospital. A top down approach: cost analysis for cardiology cases. Malaysian J Public Health..

[32] Shepard DS, Hodgkin D, Anthony Y (1998). Analysis of hospital costs: a manual for managers.

[33] Jackson T (2001). Using computerised patient-level costing data for setting DRG weights: the Victorian (Australia) cost weights studies. Health Policy..

[34] Riewpaiboon A, Malaroje S, Kongsawatt S (2007). Effect of costing methods on unit host of hospital medical services. Trop Med Int Health..

[35] Negrini D, Kettle A, Sheppard L, Mills GH, Edbrooke DI (2004). The cost of a hospital ward in Europe: is there a methodology available to accurately measure the costs?. J Health Organ Manag..

[36] Kirby KK (2015). Hours per patient day: not the problem. Nor the solution. Nurs Econ..

[37] Mølgaard E (2000). Calculation of nursing costs in relation to the DRG-system.

[38] Aljunid SM, Ali Jadoo SA (2017). Development of pharmacy service weights in the implementation of Casemix for provider payment: concepts, methods and applications.

[39] Gong Z, Duckett SJ, Legge DG, Pei L (2004). Describing Chinese hospital activity with diagnosis related groups (DRGs). A case study in Chengdu. Health Policy..

[40] Pirson M, Delo C, Di Pierdomenico L, Biloque V, Martins D, Eryuruk U, Leclercq P (2011). Analysis of the variability of nursing care by pathology in a sample of nine Belgian hospitals. BioMed Central (BMC) Health Serv Res. From 27th Patient Classification Systems International (PCSI) Working Conference.

[41] Finkler SA (2008). Measuring and accounting for the intensity of nursing care. Is it worthwhile?. Policy Polit Nurs Pract..

[42] Wilson L, Prescott PA, Aleksandrowicz L (1988). Nursing: a major hospital components. Health Serv Res..

[43] Auffarth I, Busse R, Dietrich D, Emrich H (2008). Length of psychiatric inpatients stay: comparison of mental health care outlining a casemix from hospital in Germany and the United States of America. German J Psychiatry..

[44] Addisu F, Wondfrash M, Chemali Z, Dejene T, Tesfaye M (2015). Length of stay of psychiatric admissions in a general hospital in Ethiopia: a retrospective study. Int J Ment Heal Syst..

[45] van den Oetelaar WFJM, van Stel HF, van Rhenen W, Stellato RK, Grolman W (2018). Mapping nurses’ activities in surgical hospital wards: a time study. PLoS One..

[46] O’Brien-Pallas L, Irvine D, Peereboom E, Murray M (1997). Measuring nursing workload: understanding the variability. Nurs Econ..

[47] Dall TM, Chen YJ, Seifert RF, Maddox PJ, Hogan PF (2009). The economic value of professional nursing. Med Care..

[48] Ithman MH, Gopalakrisna G, Beck NC, Das J, Petroski G (2014). Predictors of length of stay in an acute psychiatric hospital. Biosafety Health Educ..

[49] Evers S, Voss G, Nieman F, Ament A, Groot T, Lodder J, Boreas A, Blaauw G (2002). Predicting the cost of hospital stay for stroke patients: the use of diagnosis related groups. Health Policy..

[50] McGrail K, Green B, Barer ML, Evans RG, Hertzman C, Normand C (2000). Age, costs of acute and long-term care and proximity to death: evidence for 1987-88 and 1994-95 in British Columbia. Age Ageing..

